# Effects of Road Dust Particle Size on Mineralogy, Chemical Bulk Content, Pollution and Health Risk Analyses

**DOI:** 10.3390/ijerph20176655

**Published:** 2023-08-26

**Authors:** Dídac Navarro-Ciurana, Mercè Corbella, Daniel Meroño

**Affiliations:** 1 Departament de Geologia, Facultat de Ciències, Universitat Autònoma de Barcelona (UAB), Edifici Cs s/n, 08193 Bellaterra, Spain; merce.corbella@uab.cat (M.C.); daniel.meronog@autonoma.cat (D.M.); 2 Grup MAiMA, SGR Mineralogia Aplicada, Geoquímica i Hidrogeologia, Departament de Mineralogia, Petrologia i Geologia Aplicada, Facultat de Ciències de la Terra, i Institut de Recerca de l’Aigua (IdRA), Universitat de Barcelona (UB), c/ Martí i Franquès s/n, 08028 Barcelona, Spain

**Keywords:** road dust, size fractions, potentially toxic elements, pollution, health risk

## Abstract

Because of the rising environmental and health concerns associated with atmospheric pollution caused by potentially toxic elements (PTEs), several road dust studies have been performed across the world in recent decades. This paper illustrates the effects of particle size on the PTE contents, mineralogical composition, environmental pollution and health risk assessments in road dust from Barcelona (Spain). The samples were sieved into five size fractions ranging from <45 to 500–800 µm. Although the major mineral contents (tectosilicates, phyllosilicates, and carbonates) were profuse in all fractions, the identified inhalable PTE particles (e.g., Fe, Cr, Cu, Zn, Ni, and REE), with size < 10 µm, were more pervasive in the finest fraction (<45 μm). This is consistent with the concentrations measured: the finest fractions were richer in PTEs than the coarser ones, resulting in a direct correlation with the enrichment factor (EF_x_), geo-accumulation (I_geo_), and non-carcinogenic (HI) and carcinogenic (CRI) values. I_geo_ and EF_x_ values can be appropriate tracers for some common elements (e.g., Zn, Sb, Sn, Cu, and Cr), but they do not seem adequate for anthropogenic particles accumulated at concentrations similar to the geogenic background. Overall, the HI and CRI values obtained in Barcelona were acceptable, reflecting no serious health impacts in the study area, except for Cr. Our results suggest that fine dust particles are a more suitable fraction to conduct pollution and health risk assessments than coarser ones, although the EF_x_, I_geo_, HI, and CRI threshold values should be redefined in the future to include all emergent pollutants as well. In summary, monitoring programs should include at least the road dust evaluation of <45 µm particles, which can be performed with a simple sieving method, which is both time- and cost-effective.

## 1. Introduction

Ambient particulate matter (PM) pollution is one of the global challenges plaguing modern society [1]. PM that is deposited on roads, usually called “road deposited sediments”, “street dust”, or “road dust”, are significant pollutants of the urban environment because it contains high levels of poisonous metals and organic contaminants [2,3,4]. Road dust particles function as carriers of potentially toxic elements (PTEs) [5]. They are persistent and non-biodegradable, and they often tend to remain in the environment for decades after the termination of the source of the emissions [6]. Therefore, characterizing the dust particle sources and chemical and mineralogical conformations on urban road surfaces is essential for developing an appropriate pollution evaluation and designing mitigation strategies.

Road dust in the urban environment is a heterogeneous composite from multiple natural geogenic and anthropogenic sources [7,8,9,10]. Potential geogenic sources of road deposited particles include erosion of surrounding soil and local geological formations, as well as atmospheric deposition of transported geogenic particles [11,12]. Anthropogenic sources of particle pollutants in road dust include industrial emissions (power plants, coal combustion, the metallurgical industry, auto repair shops, the chemical industry, etc.) and traffic-related activities, such as tire and brake abrasion products, automotive three-way catalytic abrasion and wear, vehicular combustion exhaust, pavement wear, construction materials, road salt, road paint, and pedestrian debris [9,10,12,13,14]. Furthermore, road dust is subject to mixing processes with particles from different sources. Therefore, the characterization and determination of the source of road-deposited particles can be complex [15].

In the last two decades, a large number of studies have been carried out all over the world to determine the concentrations of pollutants in road dust, with the aim of identifying their sources and transport pathways, as well as their spatial distribution, bioavailability, and related health risks [16]. From low-income countries, where long-term exposure to traffic-generated dust has been estimated to cause 1.5 to 2 million premature deaths every year (mostly children and women) [17], to high- and middle-income countries, the awareness of the potential adverse impacts of dust on health has increased in recent decades. The components of road dust particles have been associated with multiple health effects, especially on the respiratory and cardiovascular systems, including allergy, carcinoma, asthma, chronic obstructive pulmonary disease, and emergency cardiovascular disease issues [3]. Despite the fact that the concentrations of pollutants in road dust depend on its mineralogical composition and the particle size and shape, mineralogical and morphological studies of road dust particles are scarcer than chemical bulk composition studies.

In attempts to identify metal pollutant sources and assess health risks, dust samples have been fractionated according to the particle sizes in several studies. In a review by Lanzerstorfer [18], the highest metal pollutant concentrations were found in the finest size fractions of the road dust. As demonstrated by Lanzerstorfer and Logiewa [19], the heavy metal concentration of a road dust sample with a size fraction of <37 µm can be eight times larger than the same sample with an upper size fraction limit of 2000 µm. Many road dust publications contain tables and figures comparing concentration results of road dust samples from different studies, illustrating quite different particle size distributions, sometimes ranging from 2 to 2000 µm. Although this could lead to misinterpretations [18], and efforts have been taken to standardize the optimal particle size range to be studied in road dusts, there is still no consensus among researchers. For instance, in air quality studies, road dust particles of <10 µm in size are widely used [8], whereas in stormwater runoff studies, fractions of <38 µm, <63 µm, or <75 µm are applied [20,21,22]. In addition, pollution and health risk assessment studies of road dust PTEs have used a wide range of upper particle size limit fractions: 63 µm, 100 µm, 125 µm, 850 µm, or up to 2000 µm [23,24,25,26]. The use of this wide range of road dust fraction sizes hinders the implementation of standardized and intercomparable pollution and health risk monitoring programs.

The present study aims to determine the effects of road dust particle size on the mineralogical composition, heavy metal contents (environmental pollution), and health risk assessments in order to suggest the most feasible size fraction for future implementation in road dust PTE monitoring programs. The study area chosen to illustrate the road dust particle size effect, Barcelona (northeast Iberian Peninsula), is one of the most air-polluted urban areas in Western Europe [27].

## 2. Materials and Methods

### 2.1. Study Area

**Figure 1:**
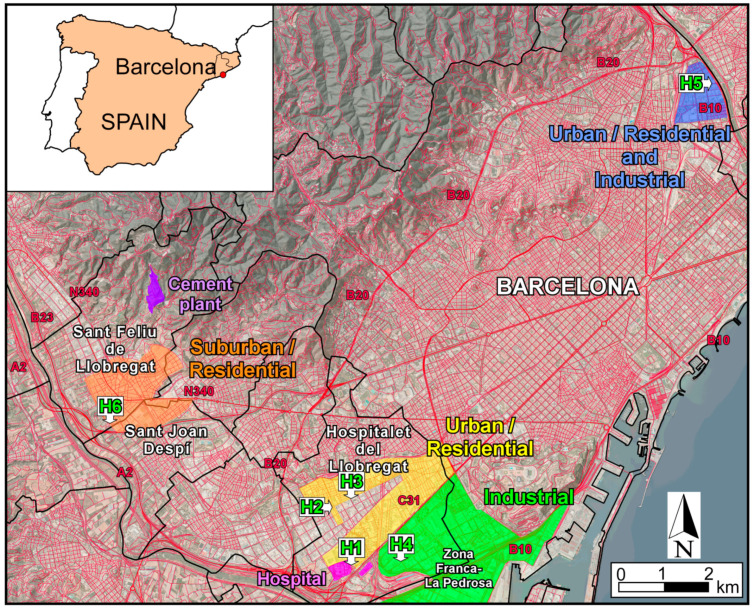
Geographical location of the road dust sampling sites (H1 to H6) in the outskirt municipalities of Barcelona (modified from [30]).

The study was carried out in the outskirts of Barcelona, with the sampling sites located also in the adjacent municipalities of Hospitalet del Llobregat and Sant Feliu de Llobregat/Sant Joan Despí (Figure 1). Barcelona is one of the European cities with the highest vehicle density (6.100 vehicles·km^−2^; [28]) and is the second most densely populated city of Spain (16.114 inhabitants km^−2^; [29]). Hospitalet del Llobregat, a city geographically adjacent to Barcelona, has also high traffic-clogged urban areas and is even denser than Barcelona (21.407 inhabitants km^−2^; [29]). In both city centers, the predominance of narrow streets (street canyons) and a dearth of green areas hinder the dispersion of pollutants. The Zona Franca-La Pedrosa is an industrial neighborhood of Barcelona located to the S of Hospitalet de Llobregat (Figure 1), which also includes various logistics and customs areas. It currently has a total surface area of 600 hectares, which represents 6% of Barcelona’s total land, and comprises more than 400 companies. Emissions from these, plus those from heavy motor traffic, coupled with the Mediterranean climate (warm and humid) and the geography of the zone, contribute to the accumulation of airborne pollutants over the city.

Sant Feliu de Llobregat and Sant Joan Despí are located to the SW of Barcelona and Hospitalet de Llobregat. Both cities have a large-scale industrial development, with a significant transport infrastructure involving trains and abundant road traffic. Since 1967, there has been a cement plant operating in Sant Feliu de Llobregat municipality (Figure 1). This plant contributes to the release of PTEs in the area, which in turn constitute an additional source of dust in the nearby roads and surrounding environment [31]. The total population under potential direct influence of the cement, industrial and transport emissions is 44,400 inhabitants, in an area of around 20 km^2^ [29].

### 2.2. Sample Sites and Dust Sampling

Road dust samples were collected in July and October of 2022 (Table 1). As wash-off from rainfall removes a portion of the road dust available on the surface [32,33,34,35,36], sampling was performed in all localities after 15 dry days. Three road types were sampled (Figure 1 and Table 1): (i) roads located in urban and suburban residential zones (H1, H2, H3 and H6); (ii) roads ubicated in land areas with intense industrial activity (H4); and (iii) roads situated in mixed residential and industrial areas (H5). Road dusting is performed in the place with the highest amount of road dust within a radius of 200 m around each sampling site, in order to collect sufficient material for chemical and mineralogical analyses.

**Figure 2:**
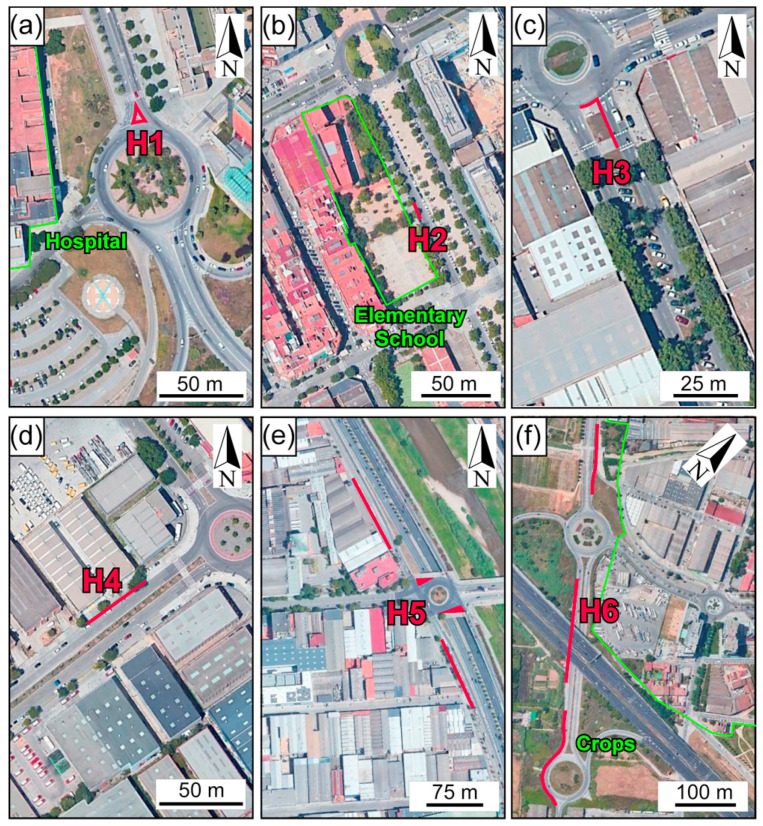
Location of sampling sites: (**a**) Hospitalet de Llobregat Mare de Déu de Bellvitge Avenue; (**b**) Hospitalet de Llobregat La Marina Boulevard; (**c**) Hospitalet de Llobregat Fabregada Avenue; (**d**) Hospitalet de Llobregat Botànica Street; (**e**) Barcelona Guayaquil Street; (**f**) Sant Feliu de Llobregat BV-2001 suburban road. The red lines indicate the swept road surface.

Approximately 27 kg of composite road dust, between 1 and 9 kg for each selected site (Table 1), were collected sweeping the selected roads with plastic bristle brushes and dustpans; the sampled material was stored in plastic bags. In Hospitalet de Llobregat, four samples (H1 to H4) were collected in different residential and industrial zones of the city. A sample was swept in the paved shoulder of a divisional island in a high-traffic avenue (H1, 41 m^2^ swept: Figure 2a and Table 1), which constitutes one of the entrances of the Bellvitge Hospital, that is perpendicular to the C-31 dual carriageway access road to Barcelona in the SW (Figure 1), one of the busiest roads of the city (avg. 70,000 vehicles per day). Additionally, in Hospitalet de Llobregat city center, a road dust sample was obtained by sweeping the paved shoulder of a medium-traffic street (H2, 30 m^2^ swept: Figure 2b and Table 1), which is located adjacent to an elementary school. Another road dust composite was collected in the paved shoulder of a medium-traffic avenue median strip (H3, 19 m^2^ swept: Figure 2c and Table 1) that crosses a residential zone of Hospitalet del Llobregat. As a representative of an industrial zone, a road dust sample was swept from the paved shoulder of a low-traffic Hospitalet de Llobregat Street median strip (H4, 87 m^2^ swept: Figure 2d and Table 1) in the Zona Franca-La Pedrosa area.

Other two samples (H5 and H6) were obtained in Barcelona and Sant Feliu de Llobregat/Sant Joan Despí limit, respectively. In Barcelona, the sample was obtained in the paved shoulder road and a clogged sewer disposed on a street concrete gutter (H5, 732 m^2^ swept: Figure 2e and Table 1), a low-traffic road that runs parallel to the B-10 dual carriageway access road to Barcelona city center in the NE. This access road is the second busiest in the city (avg. 110,000 vehicles per day). Finally, in the Sant Feliu del Llobregat and Sant Joan Despí BV-2001 suburban road, which separates the cities with a crop zone, the paved shoulder road’s median strip and a bike lane were swept (H5, 732 m^2^ swept: Figure 2f and Table 1).

### 2.3. Sample Treatment and Mineralogical and Chemical Analysis

Road dust samples were dried at 40 °C and then dry-sieved using stainless steel sieves with a range of aperture sizes of 800, 500, 250, 125 and 45 µm to obtain five subsamples per sample. These sieve size ranges were selected for comparison with soil categorization: 500–800 µm (coarse sand), 250–500 µm (medium sand), 125–250 µm (fine sand), 45–125 (very fine sand) and <45 (silt and clays). The large size fraction (subsamples of >800 µm) was discarded as it contained visible extraneous matter such as leaf litter, asphalt particles, small pieces of brick, glasses, plastics, and concrete and other debris. The other weighed subsamples were homogenized and quartered into three subsets for geochemical and mineralogical analysis.

The chemical composition of the 30 obtained subsamples was determined using Inductively Coupled Plasma-Optical Emission Spectrometry (ICP-OES) and ICP-Mass Spectrometry (ICP-MS) in accordance with the standards applied at Activation Laboratories Ltd. of Ancaster, Ontario, Canada [37]. Actlabs is a SCC GLP (Standards Council of Canada, Good Laboratory Practice) compliant facility with 45 techniques certified by ISO/IEC 17025:2017 (International Organization for Standardization/International Electrotechnical Commission) and is FDA (Food and Drug Administration) registered and inspected.

The ICP-OES method was used to determine the concentrations of major oxides (SiO_2_, Al_2_O_3_, Fe_2_O_3_, MnO, MgO, CaO, Na_2_O, K_2_O, TiO_2_ and P_2_O_5_) and selected trace elements (Ba, Be, Sr, V and Zr). In total, 1 g of sample was mixed with lithium metaborate and lithium tetraborate and fused in an induction furnace. Subsequently, the molten material was poured into a solution of 5% nitric acid containing an internal standard and mixed continuously during ~30 min until it dissolved completely [37]. The samples are run for major oxides and selected trace elements (Code 4B: [37]) on a combination simultaneous/sequential Thermo Jarrel-Ash ENVIRO II ICP or a Varian Vista 735 ICP spectrometers. Total contents should be between 98.5% and 101%. Those samples with lower results were scanned for minor and trace elements using ICP-MS. Samples with low totals, however, were automatically re-fused and re-analyzed. Low reported totals may indicate the presence of sulfidic/sulfated sulfur, as well as other elements like Li or F, which had not been scanned [37]. The detection limits (LODs) for elements analyzed using ICP-OES are shown in Table S1.

The ICP-MS method, which was used to determine the concentrations of minor and trace elements (As, Ce, Co, Cr, Cs, Cu, Dy, Er, Eu, Ga, Gd, Hf, Ho, La, Lu, Mo, Nb, Nd, Ni, Pb, Pr, Rb, Sb, Sc, Sm, Sn, Ta, Tb, Th and Y), involves melting 1 g of sample with a lithium metaborate and lithium tetraborate mixture in an induction furnace. A 0.25 g fused and dried sample aliquot is digested with a mixture of HClO_4_, HNO_3_, HCL and HF at 200 °C until fumed and is then diluted volumetrically with an aqua regia to 250 mL. The diluted samples were analyzed for trace elements (Code 4B2: [37]) with the Perkin Elmer Sciex ELAN 6000, 6100 or 9000 ICP-MS. The instrument is recalibrated every 40 samples. The LODs and upper detection limits for elements analyzed via ICP-MS are shown in Table S1.

Quality control and quality assurance (QC/QA) of chemical composition results was achieved through the analysis of reagent blank, 14 certified reference materials (CRMs, Table S2: [38,39,40,41,42,43,44,45,46]) and 17% of analytical duplicates (Table S3). The concentration of analytes in reagent blanks were measured using the same method described above in order to evaluate external metal contamination from analytical procedures. Blank samples showed no detectable analytes, indicating no contamination (Table S1). The measurement accuracy was monitored with the CRMs used (Table S4). Mean recovery rates of the CRMs major oxides and trace elements are between 89% and 118%, showing good accuracy as values are between 70% and 120% [47]. Nevertheless, recoveries for MnO, MgO, and CaO in NIST 696 (>200%) were unacceptably high due to the concentration of analytes being close to the LOD. On the other hand, mean recovery rates of the CRMs trace elements are between 95% and 104%, showing good accuracy. The precision of the analysis was calculated for each analyte as median values of the Relative Difference (RPD) between road dust sample duplicates. Median RPD values ranged from 0 to 9% for most analytes. Higher median RPD values were obtained for Pb (median RPD: 12%), Th (median RPD: 14%) and W (median RPD: 16%).

For Loss on Ignition (LOI) determination, 10 g of each dust subsample was taken and carefully poured in a clean crucible and weighed using an analytical balance (gravimetric method). The weighed samples were then placed in an electric muffle furnace and heated for 1 h at 950 °C to determine the loss on ignition. LOI, with a LOD of 0.01%, is the combined loss of volatile matter such as combined structural water (H_2_O) and carbon dioxide coming from carbonates.

The principal mineralogy of each dust subsample (30 in total) was determined via X-ray diffraction (XRD) analysis of powdered build-up solids, previously powdered to <30 µm using an agate mortar and then deposited on aluminum discs. The analyses were performed using X’Pert PRO MPD ALFA 1 (Marvel Panalytical, Almelo, The Netherlands) with a θ-θ geometry, an incident Cu-Kα, and a Cu anode X-ray tube and a PIXcel1D detector at the Servei de Difracció de Raig X of the Universitat Autònoma de Barcelona (UAB). The analytical conditions were 40 kV and 40 mA working power, with 3° divergence window and 0.05° reception window. The selected count time was 0.026° (2θ) angular step every 50 s in a 2θ angular range from 5° and 60°. The software X’Pert High Score and the PDF2 data base (ICDD: International Center for Diffraction Data) were used to evaluate the analyzed spectra. The mineral reference patterns data used are shown in Table S5 [48,49,50,51,52,53,54,55].

The mineralogical study was complemented with a particulate characterization, where representative fractions of each sample were embedded as a thin “layer” in 5 cylindrical (d = 2.5 cm) resin mounts (hereafter called monolayers). After conducting a metallographically polished of monolayers using a monocrystalline shape edges diamond abrasive paste of 1 µm, an ultrasonic bath was used to remove any remaining polishing material from the sample. To exclude contamination of the abrasive paste used, control dust samples were mounted directly on pin stubs. The samples were coated with Au-Pd alloy to improve conductivity and better image resolution after mounting the monolayers on a carbon tape attached to aluminum studs. The morphological and textural features and the semi-quantitative composition of particles in the roadside dust samples embedded in monolayers were examined at the Servei de Microscòpia at UAB using a Zeiss Merlin (Zeiss, Jena, Germany) field emission scanning electron microscope (FE-SEM) equipped with an Energy Dispersive X-ray spectroscope (EDX) to characterize the elements. The operating conditions were an accelerating voltage of 20 kV, a beam current of 1 nA and a working distance of 15 mm, with a mean EDX count time of 30 s per analysis. Acquisition of FE-SEM images allowed to determine the size distribution of road dust particles, which was based on the longest Feret diameter size and conducted using Oxford-Inca software.

### 2.4. Pollution Assessment Methodology

To evaluate road dust source and the impact of human activities on heavy metal enrichment, it is a common practice to calculate the enrichment factor of an element X (EF_x_) in the sampled material with respect to its natural abundance in the earth’s crust, according to the following equation (e.g., [56]):

(1) $${\mathrm{EF}}_{x}=\frac{{\left(X_{i}/E_{\mathrm{ref}}\right)}_{\mathrm{sample}}}{{\left(X_{i}/E_{\mathrm{ref}}\right)}_{\mathrm{background}}}$$

where X_i_ is the concentration of the element of interest and E_ref_ the reference element concentration for normalization. E_ref_ should have low variability and is used to determine the degree of metal pollution. Consequently, caution must be exercised in the choice of the reference substrate due to the great variability in trace element contents of rocks of even similar bulk composition [14]. To minimize possible error sources, the average crustal composition was not used in this study, as it may be different from the substrate of the study area; instead, the sum of Al_2_O_3_, MgO, Na_2_O, K_2_O, TiO_2_, and P_2_O_5_ concentrations reported in the Congost River basin (Table S6: [57]), which is located in the Barcelona Province, was chosen as the geochemical background. Pollution degrees according to EF_x_ are classified into five categories [56,58,59]: (i) EF_x_ < 2 means deficiency to minimal enrichment; (ii) 2 ≤ EF_x_ < 5 corresponds to moderate enrichment; (iii) 5 ≤ EF_x_ < 20 means significant enrichment; (iv) 20 ≤ EF_x_ < 40 indicates very high enrichment; and (v) EF_x_ ≥ 40 means extremely high enrichment.

The geo-accumulation Index (I_geo_) of metals is another indicator of the presence and intensity of anthropogenic contaminant deposition on road dusts. It is defined with the following equation [60]:

(2) $$I_{\mathrm{geo}}=\log_{2}\left(\frac{C_{n}}{1.5B_{n}}\right)$$

where C_n_ is the measured concentration of the elements in road dust samples and B_n_ refers to the element background value in the Congost River basin (Table S6: [57]). The constant 1.5 helps to observe natural fluctuations and detect very small anthropogenic influences of elements. Müller [60] defined seven I_geo_ classes: (i) I_geo_ ≤ 0 indicates no contamination; (ii) 0 < I_geo_ ≤ 1 refers to uncontaminated to moderate contamination; (iii) 1 < I_geo_ ≤ 2 means moderate contamination; (iv) 2 < I_geo_ ≤ 3 indicates moderate to heavy contamination; (v) 3 < I_geo_ ≤ 4 corresponds to heavy contamination; (vi) 4 ≤ I_geo_ < 5 means heavy to extreme contamination; and (vii) I_geo_ ≥ 5 indicates extremely contaminated material.

### 2.5. Health Risk Assessment Methodology

The model used in this study to identify human exposure to road dust PTEs and the associated health risks (non-carcinogenic and carcinogenic) for children and adults in different fractionated samples is based on those developed by the United States Environmental Protection Agency (USEPA: [61,62]). The model applied assumes that local residents are exposed to metals in road dust through three pathways: direct ingestion, inhalation through mouth and nose and dermal absorption of dust particles.

According to the Exposure Factors Handbook [63] and the Technical Report of USEPA [64], the equations for calculating the average daily dose (ADD: mg kg^−1^ day^−1^) of PTEs through the three exposure routes are as follows:

(3) $${\mathrm{ADD}}_{\mathrm{ing}}=\frac{C\;\times {\;\mathrm{Ing}}_{R}\times \;\mathrm{CF}\;\times \;\mathrm{EF}\;\times \mathrm{ED}}{\mathrm{BW}\;\times \mathrm{AT}}$$

(4) $${\mathrm{ADD}}_{\mathrm{inh}}=\frac{C\;\times {\;\mathrm{Inh}}_{R}\times \;\mathrm{EF}\;\times \mathrm{ED}}{\mathrm{PEF}\;\times \;\mathrm{BW}\;\times \mathrm{AT}}$$

(5) $${\mathrm{ADD}}_{\mathrm{dermal}}=\frac{C\;\times \mathrm{SA}\times \;\mathrm{CF}\;\times \;\mathrm{AF}\;\times \;\mathrm{ABF}\;\times \mathrm{EF}\;\times \mathrm{ED}}{\mathrm{BW}\;\times \mathrm{AT}}$$

where the ADD_ing_, ADD_inh_ and ADD_dermal_ are the average daily dose (mg kg^−1^ day^−1^) exposure to metals through ingestion, inhalation, and dermal contact, respectively. The detailed description of the values of exposure factors for children and adults applied to the above models are presented in Table 2.

In order to analyze the human health risks due to PTEs exposure in the road dust, the HQ (chronic hazards quotient), HI (cumulative non-carcinogenic hazard index) and CRI (carcinogenic risk index) were computed for some selected elements and pathways applying the flowing equations:

(6) $$HQ_{x}=\frac{ADD_{x}}{R_{f}D}\;$$

(7) $$HI=\sum^{\;}HQ_{x}=HQ_{ing}+HQ_{inh}+HQ_{dermal}$$

(8) CRI=LADD×SF

where x refers to the pathway considered (ingestion, inhalation and dermal), $R_{f}D$ and SF are the values of reference’s dose and chronic slope factor, respectively, and LADD (mg kg^−1^ day^−1^) is the lifetime average daily dose exposure to carcinogen metals, for cancer risk, which is expressed as

(9) $$LAAD=\frac{C\times EF}{AT\times PEF}\times \left(\frac{CR_{child}+ED_{child}}{BW_{child}}+\frac{CR_{adult}+ED_{adult}}{BW_{adult}}\right)$$

where CR is the contact frequency. In this study, CR for ingestion is the same value of Ing_R_ × CF, for inhalation route is equal to Inh_R_/PEF, and dermal pathway is expressed as CR_dermal_ = SA × AF × ABS × CF [72].

The $R_{f}D$ values for ingestion inhalation and dermal contact used for HQ and HI calculations were available for the three pathways for most of the elements considered in this study (Al, Fe, Mn, V, Ba, Cr, Co, Ni, Cu, Zn, As, Mo, Sb, and Pb) except for Sr, Tl and U, wherein the $R_{f}D$ values for inhalation were not available (Table S7). HQ ≤ 1 indicates that adverse effects are not likely to occur, and thus can be considered to have negligible hazard, while HQ > 1 suggests that there is a high probability of non-carcinogenic effects to occur [68]. To estimate the overall developing hazard of non-carcinogenic effects, it is assumed that toxic risks have additive properties. Therefore, HI value, which is the sum of HQ for different substances through each pathway, refers to the total risk of non-carcinogenic for a signal PTE [69,73]. Values of HI ≤ 1 imply that no significant risk of non-carcinogenic effects is likely to occur. On the other hand, there is a chance that non-carcinogenic effects occur when HI >1, and the probability increases with increasing HI values [65,66].

The CRI estimates values which act as a probability for an individual to develop any type of cancer through lifetime exposure to carcinogenic hazards [24,73]. In this study, the carcinogenic risk from road dust was estimated for the carcinogens including Cd, Co, Cr and Ni, Pb and As through the possible routes. Slope factors were available for inhalation pathway only for the most carcinogen elements considered in this study (Cr, Co, Ni, As, and Pb), except for As and Pb, for which slope factors for ingestion and dermal absorption pathways were also available (Table S7). The USEPA recommended that the value of CRI < 1 × 10^−6^ can be regarded as negligible, whereas CRI > 1 × 10^−4^ is likely to be harmful to human beings. Therefore, the acceptable or tolerable risk for regulatory purposes is in the range of 1 × 10^−6^ to 1 × 10^−4^ [65,66,68]. Nonetheless, other regulatory agencies use a lower threshold value of 1 × 10^−5^ [67].

## 3. Results

### 3.1. Road Dust Size Distribution

**Figure 3:**
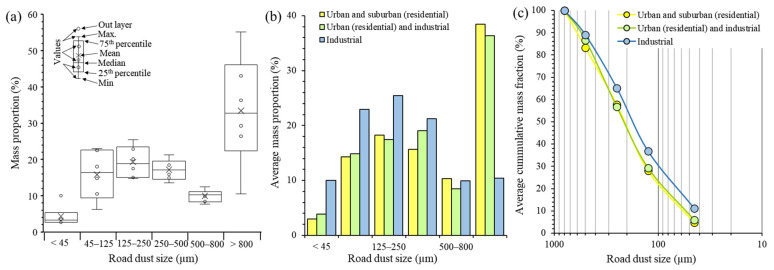
Particulate size distribution of road dusts: (**a**) size-fractional relation to mass percentage; (**b**) average size distribution of particles in road dust according to the land use type; (**c**) average cumulative mass percentage of road dust with respect to land use type.

As expected, the mass of road dust obtained per unit area, calculated using the data of Table 1, was highest in the high-traffic road (130 g m^−2^; H1), followed by areas with medium-traffic (55–66 g m^−2^; H2 and H3) and low-traffic (7–36 g m^−2^; H4, H5 and H6) volumes. But the mass collected was also dependent on the fraction size and road use (Figure 3). The mass percentages ranges of the fractions (<45 μm, 45–125 μm, 125–250 μm, 250–500 μm, 500–800 μm and >800 μm) were 2.6–10.0%, 6.2–23.0%, 14.8–25.4%, 13.5–21.2%, 7.6–12.5% and 10.4–55.2%, with mean values of 4.3%, 15.8%, 19.3%, 17.2% 10.0% and 33.5%, respectively (Figure 3a). Fractionation of bulk samples to various particle sizes revealed that the >800 μm was the dominant mass fraction of the dust from all sites, except the industrial ones (Figure 3b). Nevertheless, the size distribution of studied road dusts discarding the >800 μm mass fraction depicts maximum values in the 125 to 500 μm size range. This result is consistent with the size distribution study of other worldwide road dusts (e.g., [74]).

Samples from urban and suburban residential areas contained less mass percentage of fine particles (<45 μm; 3.0%) and more mass of coarse particles (>800 μm; 38.5%) than industrial ones or with mixed land uses (Figure 3b). Furthermore, the cumulative particle size distribution curves of road dust from industrial and urban/suburban residential zones were slightly different, although both showed similar trends (Figure 3c). Road dust contained more mass fraction percentages (less than 800 μm) in industrial zones in relation to urban and suburban residential areas and of mixed land uses. The larger size fraction of the studied samples contained high amounts of organic matter as decomposed leaf litter, except in industrial zones, which in the studied area are characterized by a scarcity of green areas with vegetation or soils. Although a larger number of samples collected in industrial and in mixed land uses would be necessary to accept their apparent difference with industrial and residential areas as statistically significative, our data still reflect a distribution dependence of road dust particles with the use of land, as it occurs in other world sites [74,75].

### 3.2. Road Dust Chemical Composition

**Figure 4:**
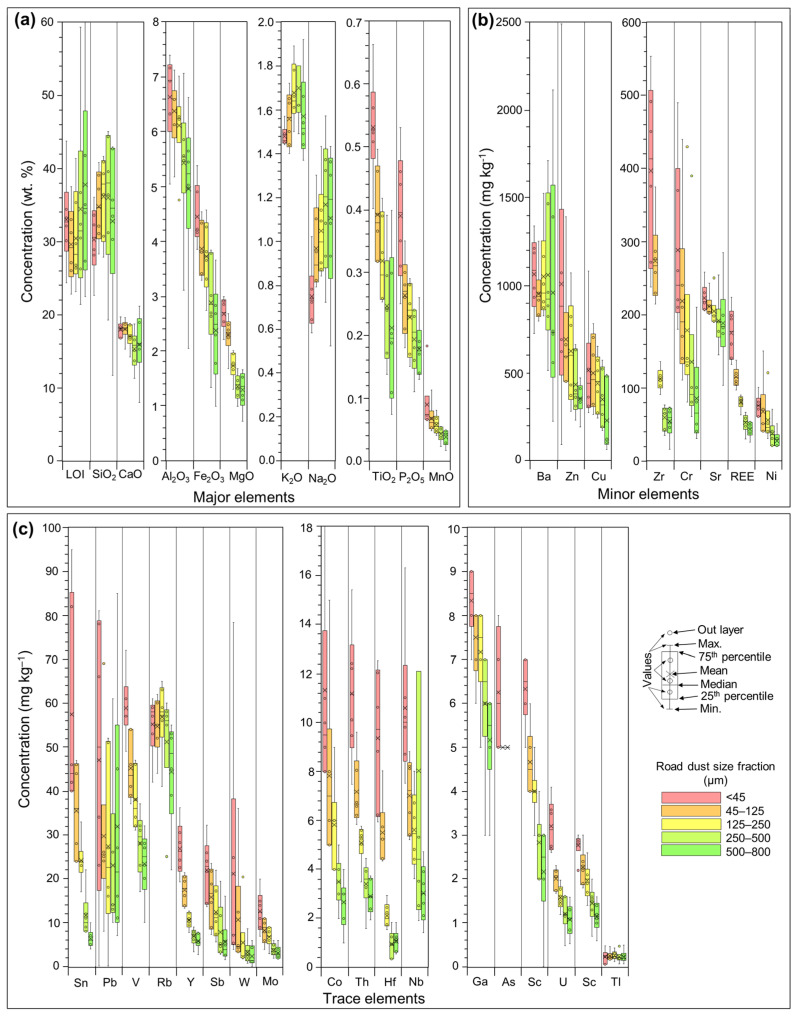
Box plots of road dust component concentrations distribution per size fraction of major (**a**), minor (**b**) and trace elements (**c**). The color bars for each element refer to the sample size fraction, with red color representing the finer parts concentrations and green color representing the coarser ones.

The concentrations of major, minor and trace elements in road dusts separated by size are shown in Table S8 and summarized in Figure 4. Overall, the highest concentration of most major, minor and trace elements were associated with the fine (<45 µm) size fraction, which is in concordance with previous worldwide case studies [75,76,77,78]. The exceptions are for K_2_O, Na_2_O and Ba. These components also present a direct correlation trend between size fraction and concentration, which is opposite to the other components. The most abundant trace metal measured (in weight) was Ba (average for all fractions: 1013 mg kg^−1^). Other trace metals that followed were Zn, Cu, Zr, Cr, rare earth elements (REE) and Ni with an average concentration value in the finest fraction of 1006.67, 511.67, 397.17, 288.33, 174.65 and 75.00 mg kg^−1^, respectively (Figure 4).

### 3.3. Road Dust Mineral Components

**Figure 5:**
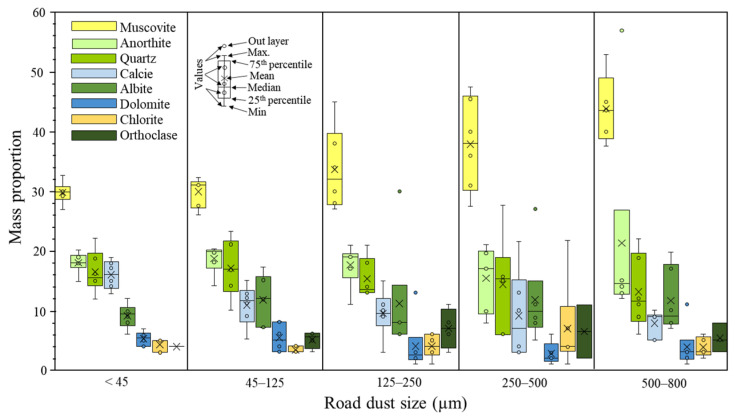
Bow and whisker plots of major mineral mass proportions respect to road dust particle sizes.

The mineralogical composition of the dust samples is shown in Table S9 and the statistical analysis with respect to size distribution for the studied road dusts is displayed in Figure 5. Regardless of the sampled site, all studied road dusts are constituted with the same major mineral components: tectosilicates, phyllosilicates and carbonates. The most abundant mineral was muscovite (KAl_3_Si_3_O_10_(OH)_2_: avg. 35%), followed by anorthite (CaAl_2_Si_2_O_8_: avg. 18%), quartz (SiO_2_: avg. 15%), albite (NaAlSi_3_O_8_: avg. 11%) and calcite (CaCO_3_: avg. 11%). Other mineral components found were chlorite (avg. 5%), dolomite (avg. 4%) and orthoclase (avg. 6%), which were present only in a few fractions of the studied samples. These mineral phases were also observed under FE-SEM-EDX, constituting the main matrix of the fractionated studied dusts.

The mass percentage of muscovite was directly proportional to grain size, whereas quartz and carbonates (calcite and dolomite) mass proportions were inversely proportional to grain size. Overall, the average mass proportion of albite, anorthite and chlorite was constant regardless of grain size. Furthermore, anomalies in the XRD background signal of the 250–500 μm and 500–800 μm fractions of the H3 sample were identified, suggesting a significant proportion of amorphous components, such as glasses. The results obtained were consistent with previously published research data (e.g., [79]).

**Figure 6:**
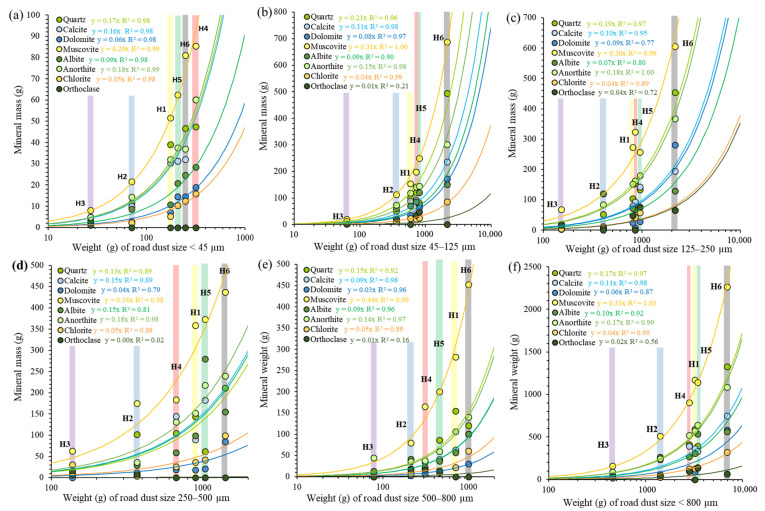
Plots of major mineral mass content (in a logarithmic scale) vs. total weight for the different fractions of road dust: (**a**) <45 µm fraction; (**b**) 45–125 µm fraction; (**c**) 125–250 µm fraction; (**d**) 250–500 µm fraction; (**e**) 500–800 µm fraction; (**f**) <800 µm fraction. Vertical color bars correspond to the different sample localities (H1 to H6) and color curves are the best fitting functions for each mineral and fraction.

It is important to note that phyllosilicate crystals tend to orientate during sample preparation, modifying the XRD intensity of reflection compared to a random orientation. This preferred orientation leads to an interference mainly with the quartz XRD peak, which commonly leads to overestimation of clay minerals and mica mass percentages and quartz underestimation (e.g., [80]). Nevertheless, this serious interference must have occurred in all samples, and thus fractionated road dust samples can be compared with each other anyway. The mass mineral content calculated and compared with the total weight of each fractionated dust sample is shown in Figure 6. Except for orthoclase, a high positive correlation of weights between mineral content and total dust for each size fractions in all sampling sites is clear. The relations between mineral mass and fraction mass are linear, although the doubly logarithmic plot appear as curves. The equations relating these two variables have higher slopes for muscovite (0.31, R^2^: 1.00) than for the quartz and anorthite (0.17, R^2^: 0.99), calcite (0.10, R^2^: 0.98), albite (0.09, R^2^: 0.92), dolomite (0.06, R^2^: 0.87) or chlorite (0.04, R^2^: 0.92) (Figure 6f).

**Figure 7:**
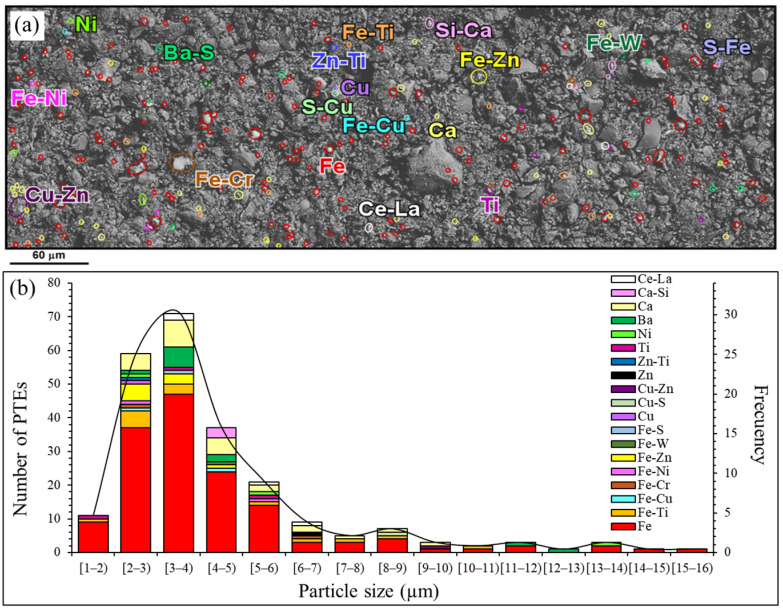
Characterization of road dust particles with diameters of less than 45 µm: (**a**) backscattered electron image identifying toxic heavy metal particles in a representative section; (**b**) histogram of PTE particle size distribution and composition determined with FE-SEM-EDX.

FE-SEM-EDX revealed that small heavy microparticles (size < 10 µm) are more pervasive in the finest (<45 μm) than in coarser dust fractions. Therefore, the microparticle sizes and compositions of the finest dust fraction (<45 μm) are more suitable for studies of heavy metal or PTE characterization of road dust than any other fraction. Figure 7 shows the mineral distribution of 234 PTE microparticles from the finest fraction of sample H1 in a surface of 120 mm^2^ (Figure 7a). Although the studied heavy metal particles diameter ranges from 1.5 to 15.3 µm, 71% of them were between 2 and 5 µm (Figure 7b). Most of the particles (63%) constituted of only Fe with sizes between 1.5 and 15.3 µm, suggesting they were native Fe or Fe-(oxi)hydroxides, and 13.2% corresponded to Fe-alloys with Cr, Ni, Cu, Ti, W and Zn with sizes of 2.3 to 10.1 µm. This abundance of Fe microparticles is consistent with the high Fe concentration in the finest road dust fractions (Figure 4).

A small number of particles (3.8%) with Ba and S, corresponding to barium sulfate as was reported in previous works (e.g., [14]), were present with diameters between 2.8 and 4.6 µm. As barium is a heavy element, it may correspond with the high overall Ba concentration obtained (Figure 4). Furthermore, Cu microparticles are less abundant (1.3%): their size ranges from 2.0 to 9.4 µm and correspond to native Cu or Cu-(oxi)hydroxide, Cu-Zn alloys and Cu sulfates or sulfides. Metallic Ni, Ti and Zn, as well as Zn-Ti alloy particles were identified with diameters ranging from 2.3 to 13.7 µm. Microparticles of Ca, which could correspond to Ca-(oxi)hydroxide and Ca-silicate (10.7%), were identified with spherical morphologies and sizes ranging from 2.7 to 12.1 µm. It is important to remark that 1.7% of heavy particles were constituted by rare earth elements (REE), mainly Ce and La.

### 3.4. Evaluation of the Road Dust Pollution

**Figure 8:**
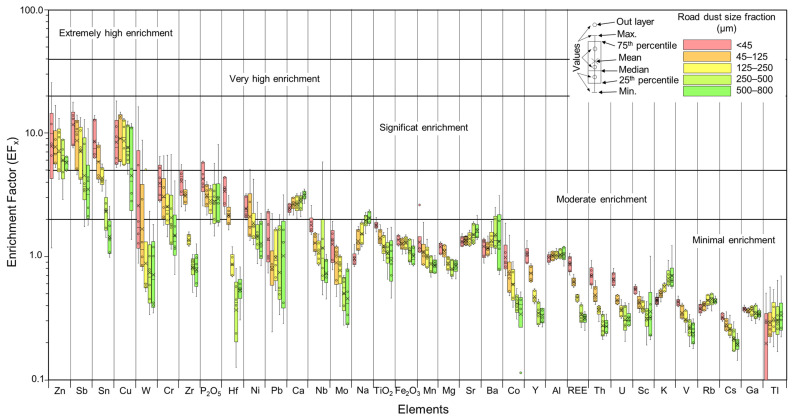
Bow and whisker plots of EF_x_ of selected elements for the different road dust particle size fractions (color codes as in Figure 4).

Enrichment factors (EF_x_) were calculated for each size fraction of road dust and it allowed for the distinction between natural sources from anthropic origins of metals. Cu was the most enriched element in the studied road dust samples followed by Zn, Sb and Sn. The average EF_x_ values for these elements in all road dust fractionated samples were between 5 and 9 (Table S10), indicating a significant enrichment and therefore a dominant anthropogenic or non-geogenic contribution. The rest of the considered metals EF_x_ average values were lower than 5, and more commonly lower than 1, suggesting that they could mainly have natural sources (Table S10). Moreover, it can also be observed that the averaged EF_x_ values for most metals were inversely proportional to particle size (Figure 8), that is, the finest dust (<45 µm) was more easily enriched and more susceptible to having anthropic origins than coarser dust (500–800 µm). Except for Zn, road dust with sizes between 500 and 800 µm showed EF_x_ values lower than 5 (Figure 8), indicating a minimal to moderate enrichment with respect to the background values and were thus considered a priori to have been mainly derived from natural sources. Overall, the enrichment factors of Zn, Sb, Sn and Cu were similar, with the EF_x_ being higher in the finest fractions (avg. Zn = 10.8; avg. Sb = 12.2; avg. Sn = 9.0; avg. Cu = 9.3) than in coarser dust (avg. Zn = 5.7; avg. Sb = 4.3; avg. Sn = 1.5; avg. Cu = 5.9) (Figure 8). Zn and Sb EF_x_ with size <45 µm was twice as large as the same dust sample with a size of 500–800 µm, whereas for Cu and Sn, EF_x_’s were three and six times higher, respectively.

**Figure 9:**
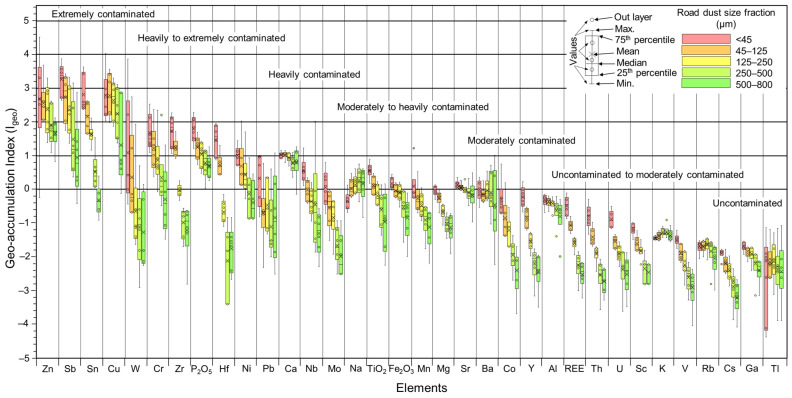
Bow and whisker plots of the geo-accumulation index (I_geo_) for selected elements in the different road dust particle size fractions (color codes as in previous figures).

The geo-accumulation index (I_geo_) values for the selected metals in terms of the various dust size fractions are presented in Figure 9. Except for Cu, Zn, Sb, Sn, Ni, Cr and Zr, and based on Muller scales [60], the average I_geo_ values for all dust sizes were negative, suggesting no contamination with respect to those elements (Table S10). Nevertheless, as with the EF_x_ values, the average I_geo_ for most metals increased with decreasing particle size (Figure 9). Overall, average I_geo_ values indicate that road dust <45 µm is moderately to heavily polluted by Zn, Sb, Sn and Cu, moderately contaminated by Cr, Zr, P_2_O_5_, Hf, Ni and Ca, unpolluted to moderately polluted by Pb, Nb, Mo, TiO_2_, Fe_2_O_3_, Mn and Sr, and unpolluted by the rest of the analyzed metals, such as Co, REE or V, among others.

### 3.5. Health Risk Characterization

#### 3.5.1. Exposure Assessment

The average daily exposure doses of dust contaminants in the Barcelona area for both children and adults (∑ADD for the three routes) are listed in Tables S11 and S12 (ADD_ing_, ADD_inh_ and ADD_dermal_). Overall, the toxic metal exposure amounts for children are one order of magnitude higher than values for adults (Table S11).

In terms of total exposure amounts, Mn, Sr, Cr, Cu and Zn were one order of magnitude higher (children: 1 × 10^−4^ mg kg^−1^ d^−1^; adults: 1 × 10^−5^ mg kg^−1^ d^−1^) than V, Ni and Pb (children: 1 × 10^−5^ mg kg^−1^ d^−1^; adults: 1 × 10^−6^ mg kg^−1^ d^−1^) and two orders higher than Co, As, Mo and U (children: 1 × 10^−6^ mg kg^−1^ d^−1^; adults: 1 × 10^−7^ mg kg^−1^ d^−1^). The maximum exposure doses for children and adults were 3.9 × 10^−2^ and 4.3 × 10^−3^ mg kg^−1^ d^−1^, respectively, both for Al in the finest fraction (<45 µm) and by Fe (children: 3.7 × 10^−2^ mg kg^−1^ d^−1^; adult: 4.1 × 10^−3^ mg kg^−1^ d^−1^; Table S11). Total metal exposure amounts for the two people groups according to the particle sizes was very different. Similar to EF_x_ and I_geo_, the daily exposure amounts of each considered PTE was inversely proportional to particle size, as these values are mainly controlled by the metal concentrations of fractionated road dust samples. The contribution of the finest road dust (<45 µm) to the total toxic metal exposure ranged from 19.1 to 38.5%, being 1.6 to 2.2 times higher than the contribution of the coarsest dust (500–800 µm), which varied from 8.6 to 19.1%.

#### 3.5.2. Non-Carcinogenic Risk of PTEs

**Figure 10:**
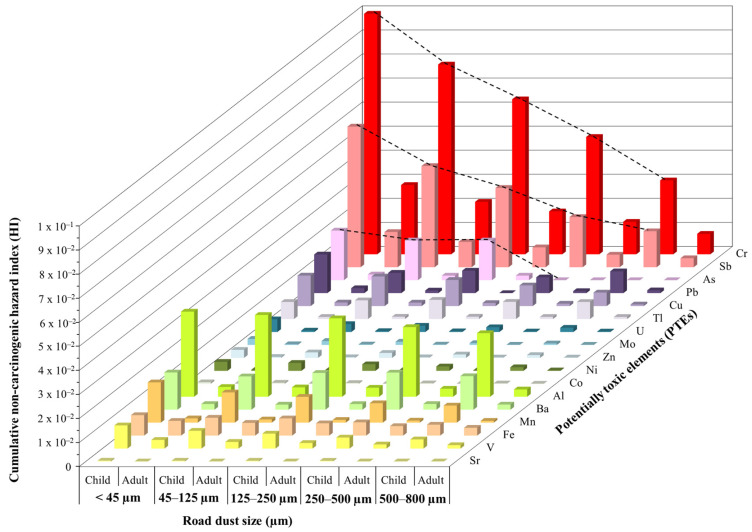
Mean hazard index (HI) values of PTEs for children and adults calculated from the different road dust particle size fractions concentrations.

The chronic hazard quotient (HQ) and cumulative non-carcinogenic hazard index (HI) values for PTEs are summarized in Table S13 and Table S14, respectively. The constituent PTEs of the non-carcinogenic risk for both children and adults are illustrated in Figure 10. HQ indices for the three exposure routes calculated for individual road dust PTEs did not exceed the acceptable level of 1 (Table S13), thus indicating negligible non-carcinogenic toxic risk [66]. In concordance with PTEs average daily exposures, the average HQ values indicate that the non-carcinogenic human health risk was higher for children than it was for adults. For children and adults, risk of Cr was the highest with 32.5%, followed by Sb, Al, Mn, Pb, Ba, Fe and Cu. Risk values of Sr and Co were the lowest, meaning they were least hazardous to human health. The contribution percentage of Sb and Al were 18.2% and 11.0%, respectively, whereas for the other considered PTEs the contribution percentages were less than 5%. Cr in the road dust, which is mainly sourced from Fe-Cr alloys (Figure 7) of the <45 µm fraction, showed the highest HI values, with a maximum of 0.18. Nevertheless, the sum of individual HQs for all PTEs considered, which determines the hazard index (HI), did not exceed the threshold value of 1 [66] for both children and adults and for all studied fractionated samples. This implies that there are no harmful health effects due to the PTE loads in the investigated road dust.

In addition, HI values for both people groups differed greatly according to particle sizes (Figure 10): (i) for Cr, Co, Mn and Sb, the non-carcinogenic health risk of particles <45 µm was 3.4 to 4.0 times higher than particles with range sizes between 500 and 800 µm; (ii) for Mn, V, Ni, Cu, Zn and U, the contribution of particles with size smaller than 45 µm to the non-carcinogenic health risk is 2.3 to 2.9-fold higher than particles of 500–800 µm; and (iii) for Al, Fe, Ba, and Pb, the finest particles contribute 1.0 to 1.9 times more to non-carcinogenic risk than coarse ones.

#### 3.5.3. Carcinogenic Risk of PTEs

**Figure 11:**
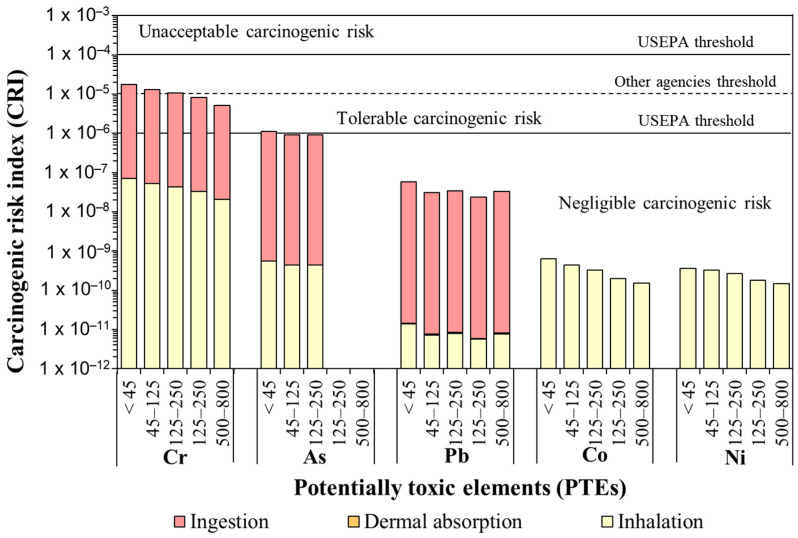
Mean carcinogenic risk index (CRI) values of selected PTEs with respect to the intake route by the different road dust particle size fractions (in µm). Limits of CRIs according to USEPA [65,66] and De Miguel et al. [67] (other agencies).

The carcinogenic risks were calculated for Cr, As, Pb, Co and Ni (Table S15), reflecting the likelihood of being infected with cancer due to road dust ingestion, dermal absorption and inhalation of these metals. Of the five selected carcinogenic metals in road dust, Cr contributed between 92.0% and 99.7% to the overall risk, followed by As. A similar observation of carcinogenic risk, mainly Cr, from road dust exposure has been reported in other cities [62,81,82]. As can be seen in Figure 11, the carcinogenic risk of Cr was in the tolerable range (between 1 × 10^−6^ and 1 × 10^−4^), whereas for Pb, Co and Ni, it could be ignored. Only the finest fraction (<45 µm) showed a tolerable carcinogenic risk of As, whereas for the coarsest fractions (>45 µm), the risk was in the limit between tolerable and negligible carcinogenic threshold. It is noticeable that all the samples fractionated to sizes lower than 45 µm showed carcinogenic risk values for Cr above the 1 × 10^−5^ level, which was deemed unacceptable by many regulatory agencies [67], but not USEPA (threshold value of 1 × 10^−6^: [65,66]). This carcinogenic risk level for Cr also decreased with the increase in particle sizes: about 66% of the total fractionated samples at 45–125 µm and less than 16.6% of samples with size ranges between 125 and 800 µm showed CRI values on the 1 × 10^−5^ order.

## 4. Discussion

### 4.1. Particle Size Effect in Road Dust Geochemical Features

Grain size has a large impact on particle hazard level [83]. Particles smaller than approximately 100–125 µm could easily be re-suspended under dynamic conditions, such as wind and vehicles running, and can enter the nose and mouth during normal breathing. Consequently, the particles with sizes <100–125 µm can be considered as health hazards. Although the number of samples collected is reduced, adding up the percentages of <45 µm and 45–125 µm fractions (Figure 3b), the highest risk to humans in Barcelona occurs in industrial parts (33.0%), followed by mixed urban/residential and industrial areas (18.6%) and urban and suburban (residential) zones (17.3%). The study by Acosta et al. [79] reported higher mass percentages of fine dust (<50 µm) on industrial zones (33.6–59.3%) than urban areas (26.2–38.8%) of Murcia city (Spain), which is in accordance with our study (Figure 3). Nonetheless, vehicle exhausts did not dominate the sources of the study road dust particles, as they are mostly smaller than 10 µm in diameter [84]. Thus, weathering soils, road erosion and pavement degradation, atmospheric particles and anthropogenic matter seem to control the main mass of dust sources as suggested by Ferreira-Baptista and De Miguel [24] and Hjortenkrans et al. [85].

The dependency of geochemical composition of road dust on their particles size (Figure 4) has significant health implications. Fine particles enriched with PTEs are more easily resuspended [24], whereas larger particles are mobilized typically through saltation and creeping [86,87], thus being able to deteriorate to smaller particles, increasing the health risk. Although the finest particles are more reactive, larger particles could also have a negative impact on the environment, as they could be transferred and mobilized to soils and be dissolved to runoff water causing pollution (e.g., [88]). In any case, given that the highest levels of PTEs were unequivocally associated with the finest fraction (<45 μm; Figure 4), a detailed mineralogic characterization under FE-SEM-EDX, specifically on heavy metal particles, was deemed necessary [89].

### 4.2. Mineralogical Composition of Fractionated Road Dust

As the major mineral content for each fraction was similar across sample sites (Figure 6), irrespective of traffic density, as commonly occurs in other world roads dusts (e.g., [90,91,92]), it was deduced that there must be a unique source unrelated to traffic or land use for the most abundant minerals present in road dust (muscovite, anorthite, quartz, albite, calcite, chlorite, dolomite, and orthoclase). Some studies confirmed that there are inputs from surrounding soils and weathered rocks to the road surfaces [90,92,93]; nevertheless, other potential anthropogenic contributions, common in all roads (cement, gravel, building materials, road paint or degraded aggregates from asphaltic pavement [94,95,96]), cannot be ruled out. These common minerals also pose human health risk. For example, the carbonatic dust with particle size of <10 µm causes eye and respiratory tract irritation [97], or the typical sharp edges of mica (i.e., muscovite) are also hazardous [79], as they cause “mica pneumoconiosis”, and in some cases interstitial pulmonary fibrosis [98]. Therefore, the composition of these particles is also an important determinant of health effects [99] and should be considered in risk assessment studies.

The essentially constant major mineral components in the different fractions of road dust (Figure 6) could be a key to remediation. A recent investigation [100] reveals the potential of road dust to be used as glass-based products for PTEs retention. As the optical, thermal, and rheological properties of glasses are directly influenced by the mineralogical composition of raw material, the uniformity of road dust content on major mineral constituents makes it a potential candidate for metal and dust remediation at an industrial scale by means of vitrification and manufacturing glasses.

The distribution and mineralogical characteristics of pollutants within the finest size fractions of road dust is quite important because this is the dust that can be resuspended. According to a study by Amato et al. [8], the contribution of road dust re-suspension to PM_10_ in Barcelona was 37%. In our study, the majority of finest identified PTE particles show size ranges between 2 and 5 µm (Figure 7) of the so called thoracic dust (<10 µm, can pass through the nose and throat, reaching the lungs); only a small portion corresponds to respirable dust (<2.5 µm), which will penetrate into the gas exchange region of the lungs [62].

The mineralogical features of most heavy particles observed in finest dust points to an anthropic origin, as previous studies have demonstrated [89,101]. For instance, barium sulfate is used as vehicles brake pad composites, similarly to metal sulfides such as covellite (Cu), pyrite (Fe), as well as chalcopyrite (Cu-Fe), sphalerite (Zn) and native elements like Cu (e.g., [9]). In addition, zinc apportionment to roads and urban areas could originate from tire wear, corrosion of safety fences and other traffic-related sources [102]. On the other hand, metallic Fe and alloys of different composition, like Fe-Ni and Fe-Cr, are typically applied as films on stainless steel (e.g., [103]), whereas the enriched microparticles with Ca could originate from concrete, which commonly are constituted by Ca-oxides and hydrated calcium silicates compounds in the SiO_2_-CaO-H_2_O system. Moreover, REE particles are probably emitted to the environment through a metastable CeZrOx segregation at the autocatalyzer under vehicles’ engine operating conditions [14]. Although road dust REE concentrations are typically in the range of geogenic values, Navarro-Ciurana et al. [14] suggested that they are a significant environmental and health hazard, and they should be included in pollution and health risk assessments. Consequently, the presence of all these heavy metal and lighter particles in the studied road dusts are probably related to the degradation of vehicle parts or other road related items and products.

### 4.3. Road Dust Pollution Evaluation and Health Risk Assessment Shortcomings

The use of I_geo_ and EF_x_ values can be appropriate tracers for some common road dust’ elements (e.g., Zn, Sb, Sn, Cu, and Cr; Figure 8 and Figure 9). Nevertheless, as discussed by Navarro-Ciurana et al. [14], elements with an anthropic origin but with low concentrations within the range of the geogenic substrate would have values of I_geo_ and EF_x_ around 1, and hence, they might be mistakenly considered as unpolluted samples. Therefore, the use of the defined I_geo_ and EF_x_ values for emergent particulate pollutants at low contents in road dust, such as REEs, might not reflect the real source nor their health risk. This limitation can be minimized through detailed mineralogical PTE studies under FE-SEM-EDX. In addition, since most of the road dust pollutants have sizes lower than 10 µm, the I_geo_ and EF_x_ calculated exclusively for this extremely finest road dusts (<10 µm) would increase considerably, probably more appropriately returning their negative impact on human health.

The children group was exposed to more road dust PTEs than adults through each of the three considered pathways (Figure 10). This result is similar to other study areas (e.g., [82,104,105,106,107]). As children in warm climate countries tend to spend a larger part of their daily lives outdoors and tend to have higher hand-to-mouth interaction during playtime [108], they are much more susceptible to PTEs health risk. Although the HI values indicate that the children of Barcelona are not under threat by road dust, it is to be noted that the non-carcinogenic health risk in children is almost nine-fold higher than that of adults. Moreover, the possibility that these metals, mainly Cr, may cause serious health effects in children (neurological and developmental disorders; [24]), through their accumulation in body tissues with time. This bioaccumulation toxicity effect does not seem sufficiently reflected in the present health risk models. Faced with the potential risks of Cr, it would be worth paying more attention to this metal concentration and take some measures to reduce it in Barcelona’ road dusts.

Overall, most of the values of carcinogenic and non-carcinogenic risk calculated in this study are within the acceptable range (Figure 10 and Figure 11). Therefore, the results reflect that exposure to toxic metals from road dust would not cause serious health impact in the study area population. However, other aspects should be taken into account. On one hand, the calculated risk values only consider a few well known PTEs, but other emergent PTEs, not often mentioned in the literature nor by competent regulatory agencies such as REE [109], which can also enter human bodies, should be included to obtain more realistic risk values. On the other hand, the road dust PTEs exposure route risk differs noticeably, and consequently, directly impacts the human risk assessment. Particle ingestion was the dominant intake route for both subpopulations, followed by inhalation and dermal contact (Figure 11, Tables S11–S14). Thus, as coarser particles (i.e., <800 µm) are easily ingested, and their mass is the highest, ingestion is the most harmful problem in relation to road dust. This exposure pattern confirms previous risk assessment results of road dust throughout the world [4,105,110].

The calculated risk (HQ, CRI) of dermal absorption seems almost negligible for adults and children alike (Figure 11 and Tables S12, S13 and S15). However, particles of 40 nm in dimeter and smaller can successfully penetrate the skin stratum corneum, whereas larger particles cannot [111]. Therefore, the exposure doses, chronic hazard quotient and carcinogenic risk index calculations through dermal absorption of the finest particle (<45 µm) of the current models may be too simple and unrealistic. Although it is a common practice to assess risk dermal absorption of road dust particles with fractionated samples at the micrometric scale [104,105,107], it should be clearly evaluated through the analysis of particles at the nanometric scale. This would require a completely different set of techniques of separation and identification and could be very time consuming.

### 4.4. Towards a Road Dust Fraction Size Standard in Monitoring Programs

Because of the clear road dust particle size effect on PTE concentrations, and consequently in the pollution and health risk assessments, it is proposed here to fractionate road dust samples and use only the finest fraction (i.e., <45 µm) in monitoring programs instead of whole bulk samples that include all size particles. The results obtained in the current study suggests that analyzing and monitoring the road dust finest fraction would be enough to assess the pollution of most common PTEs, while minimizing the related costs of analyses. There are studies that segregate particles smaller than 20 µm (e.g., [77]) using air separation or flocculation methods. Such studies have also proven the enrichment of heavy metals in the finest size fraction (1.58 ± 0.04 µm) compared to the whole road dust at <200 µm, similar to the investigation of Acosta et al. [79]. However, the techniques that allow this further fractionation to <20 µm are time and cost-consuming. This could delay monitoring programs in comparison with the sieved fractionation method used in the present study.

In alignment with Bartz et al. [89], we recommend the FE-SEM-EDX method as an affordable and highly detailed technique for obtaining information on particle size and frequency, particle shape and form, as well as their mineralogical composition and origin. Furthermore, Bartz et al. [89] suggest that the combined analysis of the chemical composition and size of atmospheric particles, carried out using an SEM-EDX analyzer, is a valuable tool for identifying potential pollutants and assessing the extent of their impact. This finding aligns with the results of our study.

In addition, the EF_x_ and I_geo_ limit values and classification should be defined by each road dust size fraction, especially for emergent pollutants with low concentration. In order to readjust the classification limits for pollution assessment according to fraction sizes, a comparison of EF_x_ and I_geo_ values calculated in the finest dust separated by air (<20 µm) with sieved dust (<45 µm) would be necessary. In any case, our data indicate that EF_x_ and I_geo_ values of fine particle dust (<45 µm) are more sensitive indicators to discriminate anthropogenic from geogenic sources, and to evaluate the pollution level for most common metals, than values of coarse particle dust (e.g., 500–800 µm: Figure 8 and Figure 9).

The cumulative non-carcinogenic hazard (HI) and carcinogenic risk (CRI) index values are mainly constrained by the road dust PTE concentrations and the reference dose ($R_{f}D$) and chronic slope (SF) factors. These values are assumed constant regardless of the road dust size fraction studied, but our results indicate that the calculated HI and CRI values of road dust PTEs through the three ingestion routes are influenced by size fraction. Therefore, a readjustment of $R_{f}D$ and SF or the HI and CRI classification limits proposed in the literature according to road dust size fraction would improve the health risk assessment evaluation. As discussed for EF_x_ and I_geo_ values inter-comparative studies between finest dust separated by air (<20 µm) with sieved dust (<45 µm) could allow to define more realistic new limits to conduct health risk monitoring programs using size fractions more efficiently in laboratories.

To sum up, monitoring programs, in city areas similar to Barcelona, should be standardized and include at least the pollution and health risk evaluation of particles with sizes lower than 45 µm. Moreover, a recalculation of either $R_{f}D$ and SF or HI and CRI specifics for the smaller particles is needed; moreover, the risk formulas should include the possible bioaccumulation time as well as the effect of non-common PTEs. The effect of nanoparticles on dermal absorption intake is another aspect to be considered for realistic estimations of road dust risk on human health.

## 5. Conclusions

Road dust mass in the urban area of Barcelona is dominated by coarse particles (>800 μm) when sampling is fractionated into five fractions: <45 μm, 45–125 μm, 125–250 μm, 250—500 μm and 500–800 μm. Regardless of the sampled site, all studied road dusts contained the same major mineral components for all sites: muscovite (avg. 35%), anorthite (avg. 18%), quartz (avg. 15%), albite (avg. 11%), calcite (avg. 11%), chlorite (avg. 5%), dolomite (avg. 4%) and orthoclase (avg. 6%), suggesting a unique source (road and traffic components). Except for orthoclase, a high positive mass correlation between mineral content and total dust for each size fractions in all sampling sites occurred, indicating a homogeneous major mineral content irrespective of land use and road traffic. In addition, the highest concentrations of potential toxic elements (PTEs) were unequivocally associated with the finest fraction (<45 μm), which is consistent with the mineralogical observation of heavy metal particles under FE-SEM-EDX. The anthropogenic PTE microparticles in the finest fraction were constituted by Fe (63%), Fe-alloys with Cr, Ni, Cu, Ti, W and Zn (13%), barium sulfate (4%), as well as native Cu or Cu-(oxi)hydroxide, Cu-Zn alloys, Cu sulfates or sulfides, metallic Ni, Ti and Zn, Zn-Ti alloys, Ca-(oxi)hydroxide, Ca-silicate, and Ce-La particles.

The calculated enrichment factor (EF_x_), geo-accumulation (I_geo_) and non-carcinogenic (HI) and carcinogenic (CRI) index values indicate that the finest road dust fraction is more dangerous for the environment and human health. Moreover, according to this study, samples with the highest risk to humans in Barcelona occur in industrial parts (33.0%), followed by mixed urban and industrial areas (18.6%) and urban and suburban zones (17.3%). The I_geo_ and EF_x_ values can be appropriate tracers for some common road dust elements (e.g., Zn, Sb, Sn, Cu, and Cr), but not for all. Overall, the HI and CRI values of Barcelona dusts were found according to the formulas of the United State Environmental Protection Agency, reflecting no serious health impacts in the study area. Nevertheless, Cr contributed between 92.0% and 99.7% to the overall risk. It is noticeable that all the samples fractionated to sizes < 45 µm showed carcinogenic risk values for Cr above the E-05 level, which is deemed unacceptable by many other regulatory agencies.

The results presented herein suggest that fine particle dust (<45 µm) is more suitable to conduct pollution and health risk assessments than coarser ones. Moreover, monitoring programs are suggested to rethink the EF_x_, I_geo_, HI and CRI thresholds or formulas to consider the effect of road dust particle size.

## Figures and Tables

**Table 1:** Samples and sampling sites.

Sample	Date	Geographical Coordinates ^1^		Altitude (masl) ^2^	Area Type	Road and Sample Locations	Area Swept (m^2^)	Sample Weight (g)
		Easting	Northing					
H1	15 July 2022	425,328	4,577,568	7	Urban/residential	Hospitalet de Llobregat Mare de Déu de Bellvitge Ave. Paved shoulder of a divisional island near to Hospital	41	5662
H2	15 July 2022	424,994	4,578,775	8	Urban/residential	Hospitalet de Llobregat La Marina Blvd.Paved shoulder adjacent to Elementary School	30	1998
H3	15 July 2022	425,351	4,579,051	8	Urban/residential	Hospitalet de Llobregat Fabregada Ave. Paved shoulder of a median strip	19	1020
H4	16 July 2022	426,418	4,577,595	6	Industrial	Hospitalet de Llobregat Botànica St. Paved shoulder	87	3150
H5	27 October 2022	433,650	4,588,295	14	Urban/residential and industrial	Barcelona Guayaquil St. Paved shoulder and clogged sewer	732	5438
H6	29 October 2022	419,948	4,580,789	17	Suburban/ residential	Sant Feliu de Llobregat BV-2001 carriageway Paved shoulder and bike lane near to crops	945	9455

^1^ WGS84, UTM zone 31; ^2^ masl: m above sea level.

**Table 2:** Values of exposure factors for heavy metals doses for children and adults.

Factor	Description	Unit	Children	Adults	References
C	Concentration of metals in road dusts	mg kg^−1^			Current study
Ing_R_	Ingestion rate of road dust	mg day^−1^	200	100	[65,66]
EF	Exposure frequency	days year^−1^	27	27	[67]
ED	Exposure duration	years	6	24	[65,66]
BW	Average body weight	kg	15	70	[62,68]
AT	Average time	days	365 × ED	365 × ED	[68]
CF	Conversion factor	kg mg^−1^	1 × 10^−6^	1 × 10^−6^	[67]
Inh_R_	Inhalation rate of road dust	m^3^ day^−1^	7.63	12.8	[65,69]
PEF	Particular emission factor	m^3^ kg^−1^	1.36 × 109	1.36 × 109	[65,66]
SA	Surface area of skin exposed to road dust	cm^2^	1600	4350	[69]
AF	Skin adherence factor	mg cm^−2^	0.2	0.7	[70,71]
ABF	Absorption factor (Dermal)	unitless	0.001	0.001	[67]

## Data Availability

All relevant data within the manuscript are available from the corresponding author upon reasonable request.

## Supplementary Materials

The following supporting information can be downloaded at https://www.mdpi.com/article/10.3390/ijerph20176655/s1, Table S1: Results for analyzed standards, as reported by ActLabs Ltd.; Table S2: Certified reference materials (CRMs); Table S3: Results for analyzed duplicates, as reported by ActLabs Ltd.; Table S4: Quality control measurements; Table S5: X-ray diffraction (XRD) reference patterns data; Table S6: Average reference values for normalization [57]; Table S7: $R_{f}D$ (reference dose) and SF (chronic slope factor) reference values [61,67,69,72]; Table S8: Major, minor and trace element concentrations of the different studied road dust samples; Table S9: Mineral phase identification (wt. %) using XRD at each site; Table S10: Average EF_x_ and I_geo_ values of selected elements in road dusts from study area. Values >2 (enriched) for EF_x_ and >1 (polluted) for I_geo_ are marked in bold; Table S11: Total exposure amounts (∑ADD) of metals in urban road dust to children and adult expressed as mg kg^−1^ d^−1^; Table S12: Daily exposure amounts (ADD) of road dust PTEs to children and adult through three routes, expressed as mg kg^−1^ d^−1^; Table S13: Chronic hazard quotient (HQ) of road dust PTEs for children and adult through three routes; Table S14: Hazard index (HI) of road dust PTEs for children and adult; Table S15: Chronic hazard quotient (HQ) of road dust PTEs for children and adult through three routes.
